# Gleanings from Our Exchanges

**Published:** 1873-05-15

**Authors:** 


Progressive Muscular Atrophy.—By B. Tauber, M.D., Paducah, Ky.—Mrs. F., aged sixty-five, has sent for me, July 20th, to examine her arm and shoulder. Some physicians, in Memphis and in this city, diagnosed the case a few months ago as rheumatism; they used liniments and alkalies *ad libitum*, with no beneficial results.

She complained of loss of strength in the arm and forearm, hand, and shoulder; severe pains, simulating those of neuralgia, in the parietic muscles; slight twitching of separate bundles of muscular fibres; locomotion in any direction lost; and showing distinctly in the hand the "*main en griffe*" of Duchenne.

On examination I found, as usual in this disease, the thenar and hypothenar eminences almost disappeared; the skin was hanging over them in loose folds; the muscles of the arm and forearm atrophied — unable to perform the complex movements; the deltoid muscle and shoulder flattened; also the trapezius and scapular muscles.

As the symptoms are so well marked, I diagnosed the case "progressive muscular atrophy," and applied fifteen Smee's cells to the spinal cord and to the sympathetic nerves; to the cord ten cells for about five minutes every alternate day. I also applied the induced current (A. Gaiffe's) directly over the atrophied muscles, to improve the nutrition of the same. To my great surprise I noticed, after the sixth application, the patient was free from pains; the voluntary movements almost natural in any direction. The lady was able to dress the hair, and to lift the arm in any desired direction.

For internal treatment, being anæmic, gave —℞. Quiniæ sulph., ʒ ss.; tr. ferri. chloridi, ʒ iii.; strychniæ sulph., gr. ss.; syr. simp., ℥ jss.; ol. anisi, gtts. iii.; M. S.—A teaspoonful three times a day after meals.

Also ordered to use the interrupted current every other or third day. The lady since in good health; the arm increased in fullness, free of pains, and she is well satisfied.—*The Cincinnati Medical News.*

Carbazotate of Ammonia in the Treatment of Malarial Poisoning. — Dr. Beaumetz brought (in a recent paper before the *Societe de Therapeutique de Paris*) the above named substance to the notice of the profession. He related the successful employment of this salt by Braconnot, Calvert, Aspland, Bell, Manopa, etc., gave the results of six cases treated by himself, and of various experiments carried on upon animals and man. Like quinine, it diminishes the strength of the pulse, brings on heaviness, cephalalgia, and even delirium, and is eliminated by the kidneys. From his experiments and cases, Dr. Beaumetz draws the following conclusions : Carbazotate of ammonia is very efficacious in intermittent fever ; the suppression of the paroxysms may be obtained by the use of one-third to two-thirds of a grain daily, given in three doses; the drug never has any bad effect, and seems to be better tolerated than sulphate of quinine, though its physiological action is very similar.—*London Lancet.—The Detroit Review of Medicine.*

